# Screening versus multidimensional assessment of symptoms and psychosocial distress in cancer patients from the time of incurability

**DOI:** 10.3389/fonc.2023.1002499

**Published:** 2023-01-26

**Authors:** Stefanie Solar, Johannes Wieditz, Florian Lordick, Anja Mehnert-Theuerkauf, Karin Oechsle, Birgitt van Oorschot, Michael Thomas, Thomas Asendorf, Friedemann Nauck, Bernd Alt-Epping

**Affiliations:** ^1^ Department of Palliative Medicine, University Medical Center Göttingen, Göttingen, Germany; ^2^ Department of Medical Statistics, University Medical Center Göttingen, Göttingen, Germany; ^3^ University Cancer Center Leipzig, University Hospital of Leipzig, Leipzig, Germany; ^4^ Department of Medical Psychology and Medical Sociology, University Hospital of Leipzig, Leipzig, Germany; ^5^ Palliative Care Unit, Center of Oncology, University Hospital of Hamburg-Eppendorf, Hamburg, Germany; ^6^ Interdisciplinary Center for Palliative Medicine, University Hospital of Würzburg, Würzburg, Germany; ^7^ Thoraxklinik and National Center for Tumor Diseases at Heidelberg University Hospital, Heidelberg, Germany; ^8^ Translational Lung Research Center Heidelberg (TLRC-H), Member of the German Center for Lung Research (DZL), Heidelberg, Germany; ^9^ Department of Palliative Medicine, University Hospital of Heidelberg, Heidelberg, Germany

**Keywords:** multidimensional assessment, screening, cancer patients, palliative medicine, quality of life

## Abstract

**Objective:**

Previous symptom prevalence studies show a diverse spectrum of symptoms and a large diversity in symptom intensities in patients being just diagnosed as having incurable cancer. It is unclear, how physical symptoms and psychosocial burden should be recorded in order to determine the variable need for palliative care and further support. Therefore, we compared two different strategies for detecting physical symptoms and psychosocial burden of patients with newly diagnosed incurable cancer and their effects on the further course of the disease.

**Methods:**

SCREBEL is a controlled, randomized, non-blinded, longitudinal study of the research network of the Palliative Medicine Working Group (APM) of the German Cancer Society (DKG). We compared: a less complex repeated brief *screening* for symptoms and burden in patients using the NCCN Distress Thermometer and IPOS questionnaire versus a multidimensional comprehensive *assessment* using the FACT-G and their entity-specific questionnaires, the PHQ4 scales, SCNS-34-SF, IPOS and NCCN Distress Thermometer. The primary study endpoint was quality of life (QoL), measured using FACT-G, after six months. Secondary study endpoints were QoL by using evaluation of secondary scores (NCCN DT, IPOS, PHQ4, SCNS-SF-34G) at time 6 months, the number of hospital days, the utilization of palliative care, emergency services, and psychosocial care structures. To assess effects and differences, multiple linear regression models were fitted and survival analyses were conducted.

**Results:**

504 patients were included in the study. 262 patients were lost to follow-up, including 155 fatalities. There were no significant differences between the low-threshold *screening* approach and a comprehensive *assessment* with respect to symptoms and other aspects of QoL. Using the IPOS, we were able to measure an improvement in the quality of life in the low-threshold *screening* arm by a decrease of 0.67 points (95%-CI: 0.34 to 0.99) every 30 days. (p<0.001). Data on the involvement of emergency facilities and on supportive services were insufficient for analysis.

**Conclusion:**

A comprehensive, multidimensional *assessment* did not significantly differ from brief *screening* in preserving several dimensions of quality of life. These findings may positively influence the implementation of structured low-threshold screening programs for supportive and palliative needs in DKG certified cancer centers.

DRKS -No. DRKS00017774 https://drks.de/search/de/trial/DRKS00017774.

## Introduction

There is abundant evidence from clinical studies that various quality of life (QoL) parameters and the implementation of patient’s goals of care and preferences (1–3) may be improved by an early (timely) inclusion of a palliative care perspective in patients suffering from incurable cancers (4–8).

Here, stage-dependent approaches (for instance, all distantly metastasized/incurable/stage IV patients should be addressed) concur with red-flags concepts (for instance, patients with typically burdensome cancer entities, with malnutrition or frailty should be addressed) (9, 10).

In an epidemiologic study, physicians estimated that 15.8% of all cancer patients who are discharged from hospital would require further palliative support (11). Other studies demonstrated a high variance in physical and psychosocial symptoms of patients with newly diagnosed incurable cancer (12, 13), which can be detected *via* assessment tools (13, 14).

An *assessment* is able to capture various symptoms and burden of patients and thus detect the need for care, which can improve the patient’s QoL and health (14). A repeated and brief, low-effort (in this paper defined as “low-threshold”) *screening*, however, appears to enhance symptom capturing and can improve quality of life and even overall survival (15, 16). All these findings suggest the usefulness of a *screening* or *assessment* approach in order to gather these symptoms and needs in a timely and structured manner. (6, 7).

The structural policy developments in Germany, for example in the context of the certification process of cancer centers of the German Cancer Society (DKG), already demand a structured screening approach for psychosocial distress by psycho-oncology. They suggest a similar approach for physical symptoms and other complaints that are relevant in pain therapy and palliative care (17). The “evidence-based guideline: Palliative Care for patients with incurable cancer” (4) recommends the repeated recording of physical, psychological, social and spiritual needs. It emphasizes the need for information on palliative issues for incurable cancer patients. However, a standard on how to most effectively capture these symptoms and needs is still missing.

In other fields of medicine, a low-threshold and easy-to-perform *screening* (like screening for psychosocial distress in psycho-oncology) competes with comprehensive, multi-dimensional *assessment* strategies (like formal baseline assessments in specialized palliative care or pain therapy, for instance). The low-threshold *screening* is a brief strategy to identify potential physical symptoms and psychosocial stress in patients, while an *assessment* is a comprehensive, multi-dimensional recording and evaluation of the patient’s symptoms and distress by using various questionnaires. A low-threshold *screening* may thus be preferred due to its resource-saving properties in patients and health care providers. However, a multi-dimensional *assessment* may suggest a more differentiated view on the complexity of physical symptoms and psychosocial distress in advanced stages of disease, even if it takes much more time and attention of patients and personnel.

Therefore, we aimed to evaluate in our SCREBEL (Screening versus multidimensional assessment of symptoms and psychosocial distress in cancer patients from the time of incurability) study the impact of two different recording strategies on QoL, the inclusion of palliative care and psychosocial support structures, emergency care structures and hospitalizations, in relation to the remaining survival time.

## Material and methods

### Study population

**Figure 1:**
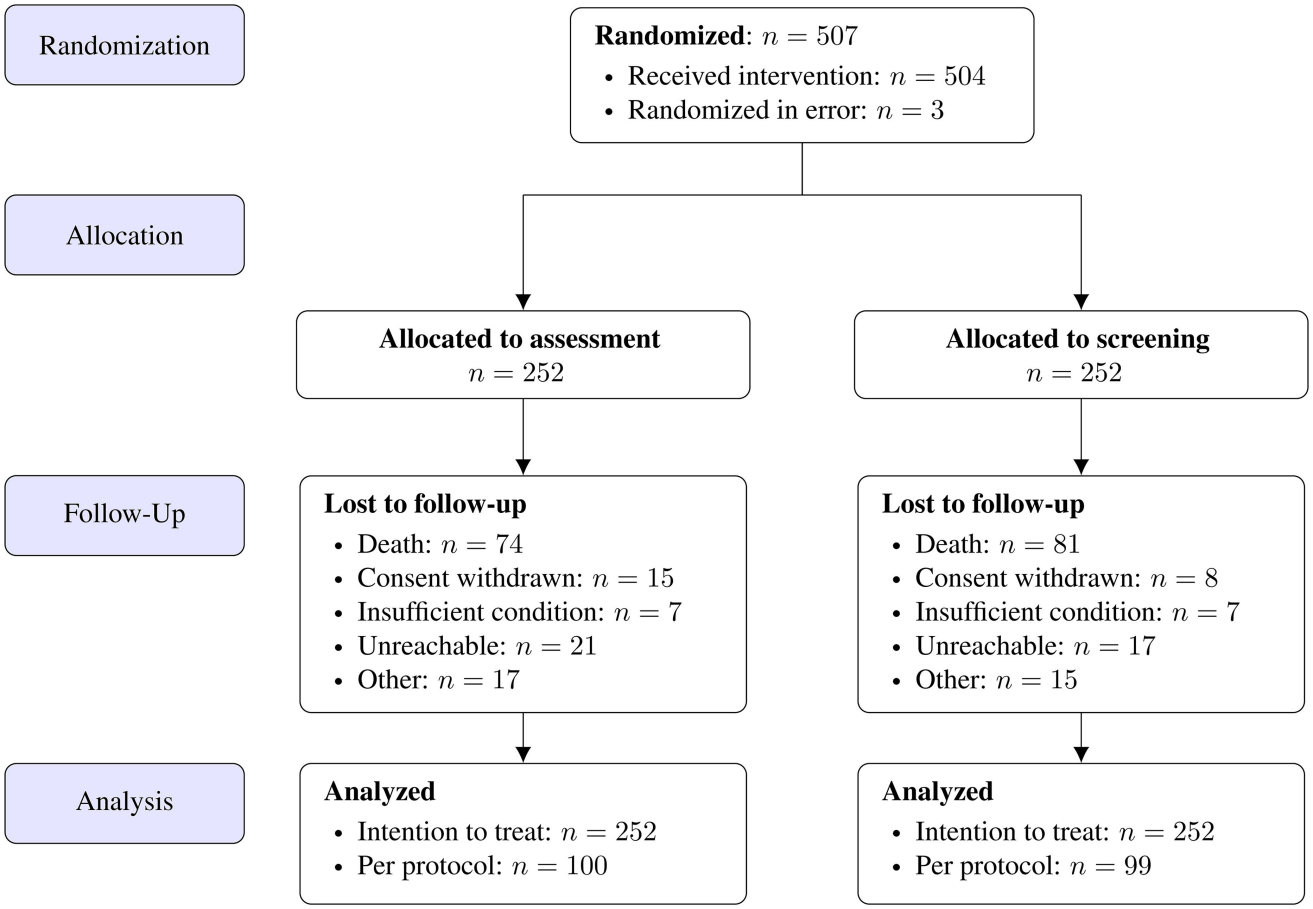
CONSORT flow diagram of patient randomization, allocation, follow-up and study analysis.

There were 24 study sites recruited by the research network of the Palliative Medicine Working Group (APM) of the DKG. In these cancer centers patients over 18 years with solid tumor entities were identified at the time of diagnosis of incurability (i.e., prior to initiation of palliative anti-cancer therapy and according to the definition of incurability as established in the former APM study (13)) *via* outpatient clinics, oncological wards or multidisciplinary tumor boards by treating physicians. Non-compliance and age being under 18 years were the only exclusion criteria. After study inclusion, the PI (Göttingen) assigned the patients to the two intervention arms (low-threshold *screening* versus comprehensive *assessment*) by using block randomization, stratified by center and tumor entity at a ratio of 1:1 (**Figure 1**). Within this study, patient intervention took place only *via* conducting surveys. The patients were requested to complete simple screening surveys autonomously on regular follow-up visits to their disease.

A population of 504 patients was estimated to be recruited for this study including an expected dropout rate of 20%. For the primary endpoint in the SCREBEL study we expected a standard deviation of 17.26 based on the experience of the former APM study (12, 13). Thus, a non-parametric test on differences between groups regarding FACT-G with a two-sided significance level α = 5% yields a power of 80% if differences are at least 5 points, which represents a clinically relevant difference (18).

### Outcome definitions

As the physicians were advised to include the results from the latest assessment into their treatment decision in order to improve the patient’s QoL we chose QoL as the primary endpoint. Quality of life was assessed using the following questionnaires:

The **FACT-G (**Functional Assessment of Cancer Therapy) (19) is a 27-item questionnaire designed to measure four domains of QoL in cancer patients: Physical, social, emotional, and functional well-being. The items are measured on a five-point Likert-scale from 0 (not at all) to 4 (very much). The score is the sum of all items and ranges from 0 to 108. The higher the score, the better the QoL.

The **NCCN Distress Thermometer** (National Comprehensive Cancer Network distress thermometer) (20) is a validated, widely used screening measure. The screening contains a single‐item visual numeric scale ranging from 0 (“no distress”) to 10 (“extreme distress”) to quantify the global level of distress experienced in the past week. A higher score indicates a higher distress and thus a lower QoL (21).

**IPOS** (Integrated Palliative Care Outcome Scale) (22) is a 10 question survey developed to measure palliative care needs of patients and their families. The questions address how limited the individual is due to the symptoms rather than the severity of the symptoms themselves. Of all questions, only the questions 2 (with again 10 subitems) and 3-9 contribute to the overall score, resulting in 17 contributing items. All items are measured on a five-point Likert scale (0 to 4). The IPOS is the sum of the 17 items mentioned above, thus ranging from zero to 68. A higher IPOS score indicates a lower QoL. Moreover, in the presence of at least two questions answered with “3” or at least three questions answered with “2”, further exploration and medical treatment is recommended.

**PHQ-4** (Patient Health Questionnaire) (23) is a four item questionnaire addressing a patient’s psychosocial condition regarding anxiety and depression. The items are measured on a four-point Likert-scale from 0 (not at all) to 3 (nearly every day). The total score is the sum of all items and ranges from 0 to 12. A total score ≥ 3 for the first two questions indicate anxiety. A total score ≥ 3 for the last two questions suggests depression. The higher the score, the lower the QoL.

**SCNS-SF-34-G** (Supportive Care needs Survey – short form 34 German Version) (24) is a 34-item questionnaire and comprises of subgroups for psychological needs (10 items), health system and informational needs (11 items), physical and daily life needs (5 items), patient care and support needs (5 items) and sexuality and other problems (3 items). The items are measured on a five-point Likert scale separated into two classes: no need (scale 1-2) and some need (scale 3-5). The overall score used for evaluation is the sum of all items of the questionnaire. High SCNS-SF-34-G scores indicate the need for more support for patients. The version used is a modified version of the SCNS-SF-34-G including only 25 questions, thus the score ranges from 25 to 125.

### Analysis populations

Analysis of participants was done in accordance with the ICH E9 guidelines for data analysis considerations (25). All randomized patients were considered as part of the intention-to-treat population. Participants of the *assessment* group were considered as per-protocol if baseline intervention took place. For the *screening* group we required at least 2 additional interventions between baseline and 6 months visit. Moreover, we require for the per-protocol population that the final visit took place 6 months after their inclusion ± 2 month. Sensitivity analyses revealed that the length of the timeframe around 6 months allowed for protocol adherence had no relevant impact on our results.

### Trial design

We performed a multicenter, controlled, randomized, non-blinded, longitudinal study and evaluated two different strategies for capturing physical symptoms and psychosocial needs.

For this purpose, the *screening* arm screened for symptoms and distress by using IPOS (15) and NCCN distress thermometer (16) repeatedly in three to six week intervals (adjusted to usual tumor therapy application cycles and oncology presentations). We did not influence whether or how palliative care was adjusted according to the screening results. IPOS was chosen as a questionnaire in the *screening* arm, since it is a validated and widely used tool in European countries to evaluate patients’ well-being and monitor their need for care (26–28). The NCCN was selected in adherence to the preceding APM study (13).

In the *assessment* arm, an initial single comprehensive recording of several dimensions of quality of life using IPOS (15), NCCN Distress Thermometer (16), plus FACT-G (general and organ-specific) (17), PHQ-4 (18), and SCNS-SF-34-G (19) has been performed. The questionnaires were again selected in adherence to the preceding APM study (13).

The study concept was designed to detect symptoms or distress, so that support measures could be initiated promptly if required. The test results were supposed to be made apparent in the institutional clinical patient´s charts, and the resulting interventions were left to the discretion of the treating team.

After 6 months, a QoL assessment was performed in both intervention arms, again using IPOS, NCCN Distress Thermometer, plus FACT-G, PHQ-4, and SCNS-SF-34-G to compare QoL. We chose this observation period as a trade-off between observing long-term effects of the intervention and avoiding excessive dropout rates, based on the data from the previous APM study (13) where data was sufficiently available after 6 months but not after 12 months, due to dropout.

In addition, study centers were asked for further data from the hospital documentation system about hospital days, emergency admissions, inclusion of specialized palliative care and other supportive services up to at most six months after start of participation.

General patient data and case report form items (CRF) were recorded in an electronic format (secuTrial).

A statistical analysis plan was written, registered and signed by the principal investigators and the responsible statistical team before analysis.

### Statistical analysis

Descriptive statistics are reported as numbers and proportions or median with corresponding range as appropriate. If not stated otherwise, tests were performed two-sided on a significance level of 5%. Parameter estimates are provided with corresponding 95% confidence intervals (95%-CI).

Primary endpoint was the FACT-G score at time t = 6 months. Differences in the relative intervention effect between study arms were tested non-parametrically using a two sample t-test for the nonparametric Behrens-Fisher problem (29). In addition, a multiple linear regression model for the FACT-G was fitted with the factor study arm (*screening* vs. *assessment*) and with additional influencing factors (tumor entity, study sites, and important prognostic baseline factors such as sex, age, personal living status and lost-to-follow-up-state). Primary and survival analysis were performed on the intention-to-treat population, and secondary and sensitivity analyses were done on the per-protocol population. Secondary endpoints at time t = 6 months (NCCN Distress Thermometer, IPOS, PHQ4, SCNS-SF-34-G) are evaluated analogously to the primary score; multiple linear regression models were fitted accordingly.

Survival rates within 6 months were estimated using Kaplan-Meier curves with additional 95% confidence bands. Comparison of the two intervention groups was performed using a log-rank test. Number of hospitalizations and mean length of stay were analyzed using negative binomial regression and zero-inflated Poisson-regression, respectively. Additionally, we fitted a Cox proportional hazards model to investigate additional factors.

For the *assessment* arm, the change of the QoL scores between initial assessment and 6 months visit was analyzed using multiple linear regression regarding the stratification factors and taking into account further covariates such as age and sex. For the *screening* arm, physical symptoms and psychosocial strains (measured *via* IPOS) were analyzed descriptively over time.

For non-parametric testing we employed a composite testing strategy and imputed missing values with the worst possible value (e.g. for FACT-G we imputed the value zero), thus associating drop-out with the least possible value for QoL. This was then interpreted as the evaluation of the full analysis set according to a worst-case approach for handling intercurrent events  (25). Additionally, we performed a complete-cases-analysis as sensitivity analysis to assess possible impairments or biases of our study results resulting from dropouts.

Differences in QoL regarding IPOS and NCCN between study arms at baseline and after 6 months, were assessed using non-parametric testing. Pre-post comparison of QoL regarding IPOS and NCCN were analysed stratified by group using paired t-testing.

For all additional analyses, missing items were imputed as proposed in the corresponding scoring guidelines. In the case of missing total scores, we imputed ten times using predictive mean matching  (30) based on study center, sex, age, entity, time of diagnosis and study arm. All data were analyzed using R version 4.2.1 (31) with additional packages mice, nparcomp, survminer for multiple imputation, nonparametric testing and survival analysis, respectively.

### Ethics and consent

The study protocol was approved by the ethics committees of all 24 study sites (PI study site no. 23/2/19) and followed the Declaration of Helsinki Ethical Principles for Medical Research. SCREBEL was registered in the German Registry for Clinical Studies (DRKS No. 00017774). Patients were included after written information, clarifications of the study and written consent.

The study was sponsored by the Innovation Funds of the German Federal Joint Committee.

## Results

The study randomized 507 patients from 24 different study centers. Three patients were randomized in error and could not be included in the study.

Of the recruited patients, 233 were female and 271 were male, with a mean age of 66.6 years. The median age was 67 years, ranging from 29 to 90 years (range 61 years). Of these patients, 314 were married*/*living in a relationship, 121 were living alone and two were living in a care facility. 67 did not state their personal living condition. Engagement of services of additional palliative care, psychosocial support and emergency structures has been recorded for only 34 patients with 16 patients documented to have frequented emergency or supportive services. Thus, this complementary data collection turned out to be insufficient for further analysis.

Of the included 504 patients, 262 were lost to follow-up after 6 months, including 155 fatalities (see **Figure 1** below).

In the study, 13 different tumor entities were included, whereby patients suffering from lung cancer were most prevalent in both study arms, followed by patients with hepatobiliary and pancreatic cancer and by skin cancers (**Table 1**).

**Table 1:** Distribution of patient demographics within the two study arms.

	Assessment (n = 252)		Screening (n = 252)	
**Age (mean ± s.d.)**	66.0 ± 11.0		67.2 ± 10.4	
Sex (n/%)*				
Female	126	50%	107	42%
Male	126	50%	145	58%
Personal living condition (n/%)*				
Married/in a relationship	148	59%	166	66%
Living alone	67	27%	54	21%
Care facility	0	0%	2	0%
No answer	37	15%	30	12%
Entity (n/%)*				
Lung cancer	68	27%	70	28%
Hepatobiliary tumors and pancreatic carcinoma	49	19%	51	20%
Skin cancer	37	15%	35	14%
Colorectal cancer	24	10%	23	9%
Head and neck tumors	23	9%	25	10%
Breast cancer	15	6%	19	8%
Endometrial and ovarian cancer	15	6%	9	4%
Gastric cancer/carcinoma	6	2%	6	2%
Urological tumors (kidneys and urinary tract)	6	2%	7	3%
Esophageal cancer	4	2%	1	0%
Brain tumors	3	1%	3	1%
Cervical and vulvar carcinoma	1	0%	1	0%
Prostate cancer	1	0%	2	1%

* Frequency (percent), s.d., standard deviation.

### Primary analysis

The primary analysis revealed no significant differences in the intervention effects on QoL (measured *via* FACT-G) between the two groups at t = 6 months, neither using the worst-case approach nor in a complete-case-analysis where the relative effects in intervention were estimated to be 0.504 (95%-CI: 0.458 to 0.549) and 0.509 (95%-CI: 0.431 to 0.587), respectively. Within the linear regression model, none of the included variables turned out to have a significant non-zero influence on QoL. (see **Table 2** below)

**Table 2:** Pairwise contrasts between *assessment* and *screening* group within multiple linear regression with respect to different questionnaires (first column), cf. also the outcome definitions section.

Questionnaire	Estimated difference (Assessment – Screening)	95%-CI	p-value
FACT-G	0.728	[-2.601; 4.058]	0.667
NCCN DT	0.169	[-0.645; 0.982]	0.683
IPOS	1.344	[-1.924; 4.612]	0.418
PHQ4	-0.002	[-0.186; 0.182]	0.986
SCNS-SF-34	1.322	[-6.005; 8.650]	0.722

FACT-G, Functional Assessment of Cancer Therapy – General; NCCN DT, National Comprehensive Cancer Network Distress Thermometer; IPOS, Integrated Palliative Care Outcome Scale; PHQ4, Patient Health Questionnaire 4; SCNS, Supportive Care Needs Survey short form 34.

With respect to the patient’s lost-to-follow-up-state we did observe only not-significantly smaller values in QoL. Moreover, we observed slightly smaller values for patients in the *screening* group whereas male patients seemed to have slightly higher QoL than females. Regarding the influence of age, QoL seemed to be insensitive.

### Secondary analyses

In accordance with our primary analysis, non-parametric testing for differences in the intervention effect on QoL measured by the secondary scores revealed no significant results likewise, both for the worst case-approach and the corresponding sensitivity analysis. Moreover, in the linear regression models the included variables did not turn out to have significant non-zero influences on QoL even though males seemed to have a somewhat smaller stress level of -0.25 points (95%-CI: -0.47 to -0.04) measured by PHQ4 than females, p = 0.023. The tendencies of the influences of the included factors direct in the same direction as outlined for FACT-G above. The estimated differences between *assessment* and *screening* group using primary and secondary outcome questionnaires are summarized in **Table 2**.

### Survival analysis

**Figure 2:**
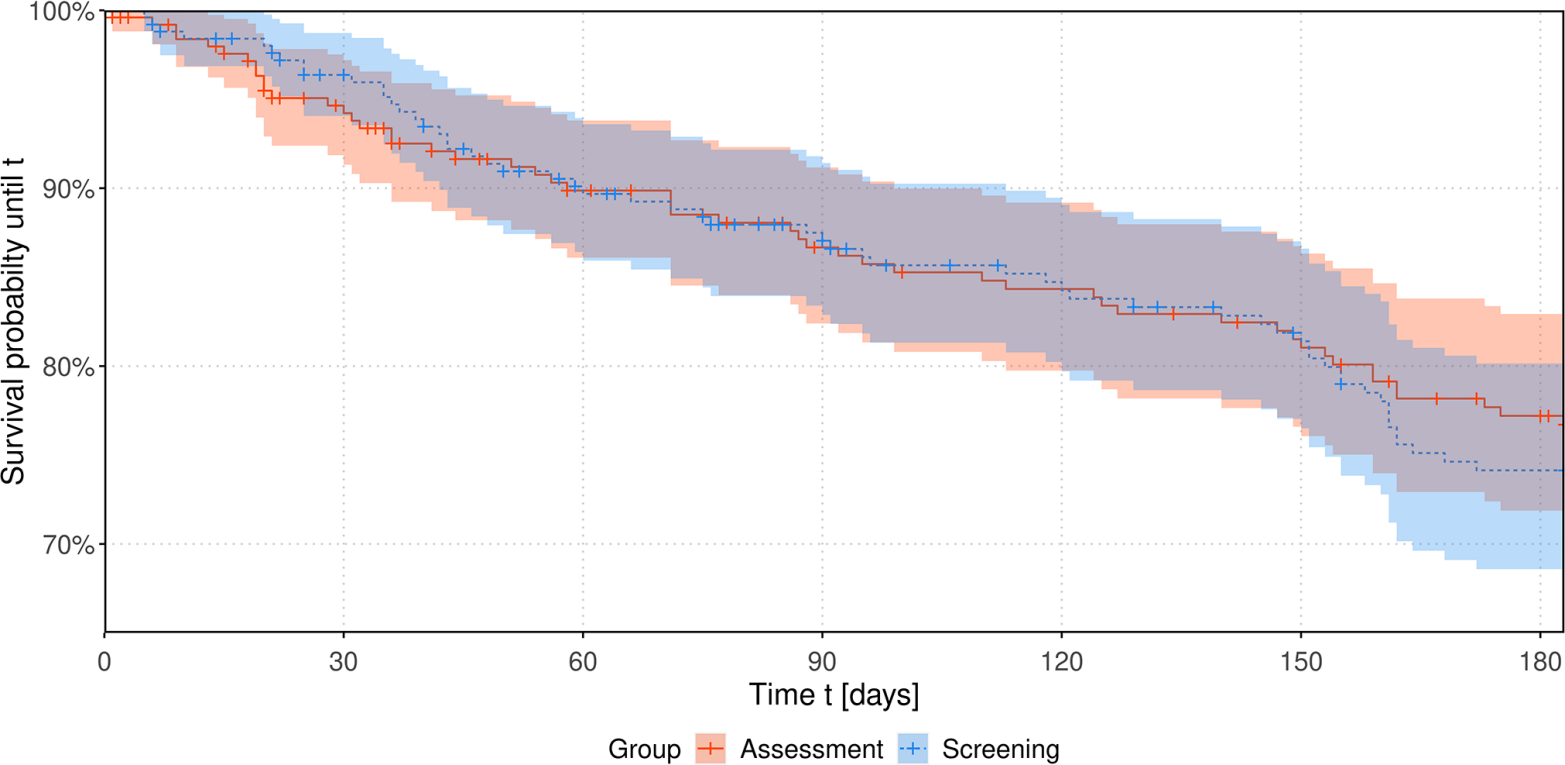
Estimated survival probabilities over time up to 6 months stratified by intervention group. Considering a Cox proportional hazards model none of the included factors study group, sex, age, study center, entity or personal living status turned out to have a significant influence on the hazard function. Affiliation to the screening group does not seem to significantly increase the hazard by a factor of 1.37 (95%-CI: 0.97 to 1.94), p = 0.073.

The course of estimated Kaplan-Meier survival curves up to six months is shown in **Figure 2**. A log-rank test for differences in survival times revealed no significant difference in the survival distribution between the two study arms, p = 0.309.

Within regression modelling, the probability of hospital admission within 6 months for the *assessment* and the *screening* group were estimated as 51% (95%-CI: 45% to 58%) and 61% (95%-CI: 55% to 67%), respectively, and turned out to differ significantly, p = 0.033. Given a hospital admission, the length of stay in *assessment* and screening group was estimated as 7.2 (95%-CI: 6.9 to 7.6) and 8.0 (95%-CI: 7.6 to 8.3) days, respectively, being significantly different (p = 0.003). The number of hospitalizations within 6 months was estimated to be 0.38 (95%-CI: 0.30 to 0.48) in the *assessment* group and 0.51 (95%-CI: 0.41 to 0.64) in the *screening* group. The difference was not significant (p = 0.07).

### Additional analyses on primary and secondary endpoints

In a linear regression model for FACT-G within the *assessment* group, none of the explaining factors age, sex and visit time (baseline vs. six months) turned out to have a significant influence. The difference in FACT-G after – before intervention was 2.2 (95%-CI: -3.0 to 7.3) which turned out to be no significant increase of QoL over time, p = 0.406.

**Figure 3:**
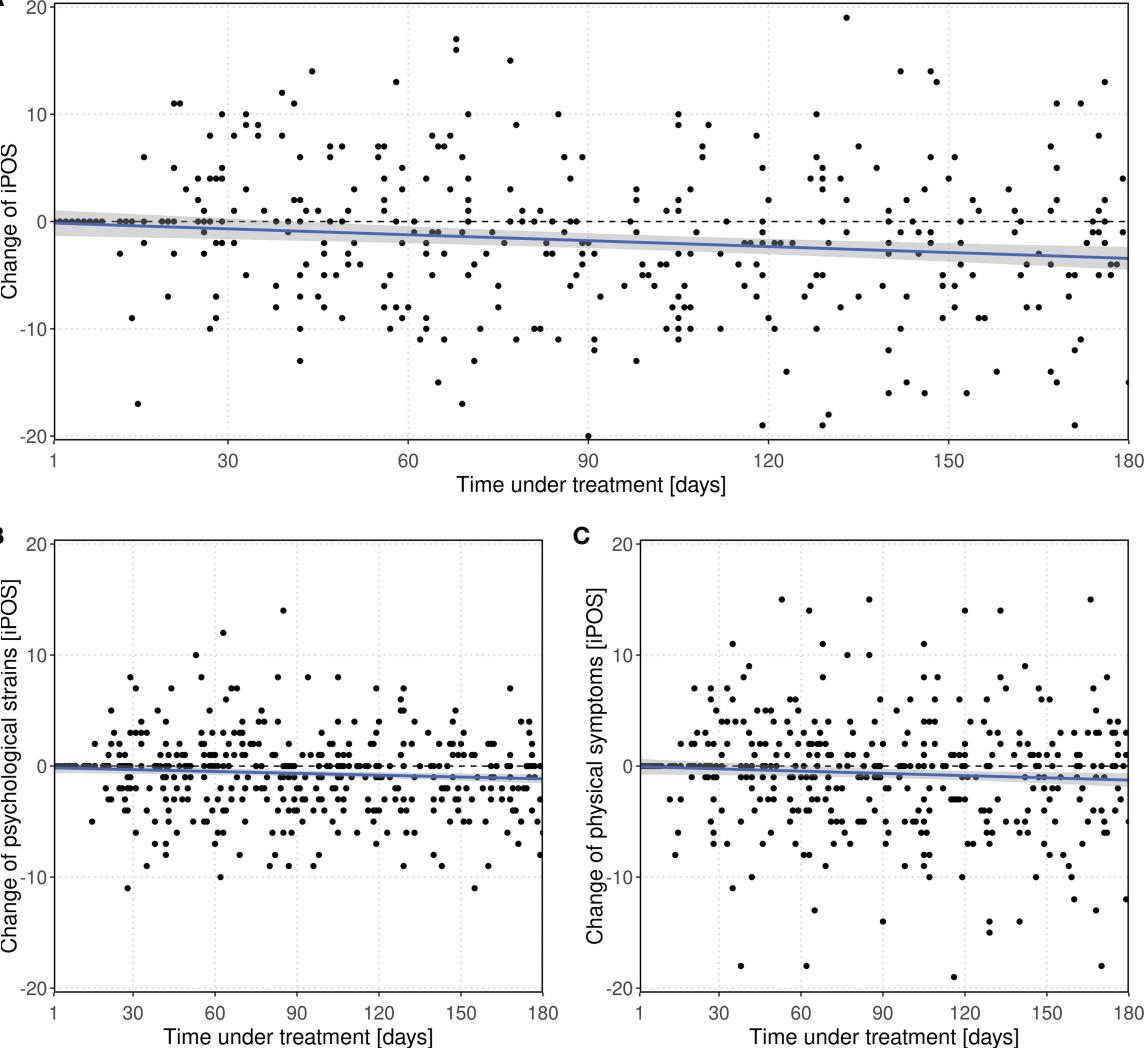
Change of **(A)** IPOS and subscores **(B)** psychological strains and **(C)** physical symptoms with respect to baseline value over time within the study within the *screening* group. Every point represents a score difference of a visit during the observation period. The dashed line at zero represents no change with respect to baseline.

Multiple linear regression of the change of IPOS with respect to baseline depending on inclusion time in the study, age and sex yielded a significant decrease over time (p < 0.001), indicating a relief of burden. Per 30 days within study inclusion, IPOS decreased by -0.54 (95%-CI:-0.84 to -0.24) points, cf. **Figure 3A**. Within the same period of time, psychological strains could be reduced by -0.16 (95%-CI: -0.28 to -0.05) and physical symptoms by -0.20 (-0.39 to -0.02), see **Figures 3B, C**, respectively.

**Figure 4:**
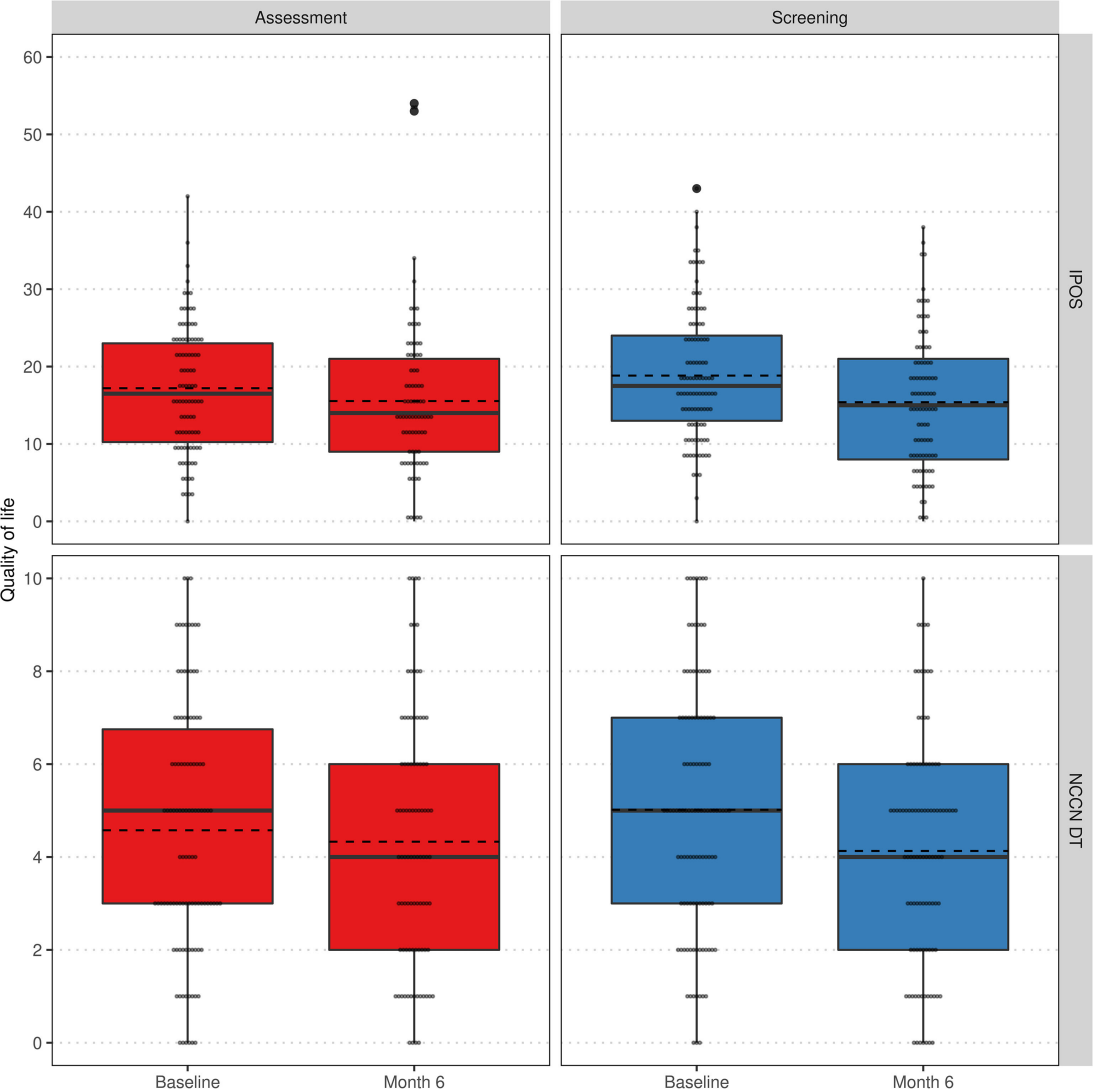
Comparison of QoL regarding IPOS (top) and NCCN DT (bottom) at baseline and 6 months visit stratified by groups *assessment* (left) and *screening* (right) within the per-protocol population. Lower values indicate a higher QoL. Individual values as small grey dots, mean within group and visit as dashed line.

Additionally, we analyzed the change of QoL regarding IPOS and NCCN DT over time within and between groups. Neither at baseline nor after 6 months, IPOS, its subscores for psychological strains and physical symptoms or NCCN DT differed significantly between *assessment* and *screening* group. In the *assessment* group, a significant improvement of QoL could only be achieved with respect to the psychological strains subscore. In contrast, the *screening* group exhibited a significantly better quality of life after 6 months regarding all considered scores (see **Table 3**). The distribution of QoL changes within study arms are visualized in **Figure 4**.

**Table 3:** Changes in mean QoL after 6 months visit with respect to baseline with corresponding 95%-CI and p-values of paired t-tests.

	Assessment	P-value	Screening	P-value
IPOS	–1.52 [–3.68; 0.65]	0.166	–3.49 [–5.42; –1.57]	<0.001
IPOS – Psychological strains	–1.19 [–2.00; –0.38]	0.005	–1.17 [–1.79; –0.55]	<0.001
IPOS – Physical symptoms	–0.54 [–1.63; 0.54]	0.321	–1.11 [–2.20; –0.02]	0.046
NCCN DT	–0.28 [–0.86; 0.30]	0.339	–0.89 [–1.39; –0.38]	<0.001

## Discussion

There were no significant differences in the improvement in QoL after a six months period between the *assessment* arm and the *screening* arm. Based on our data, both low-threshold *screening* and comprehensive *assessment* might be an appropriate strategy to record symptoms and stress for patients in order to best maintain the patients’ QoL. Since low-threshold screening saves resources and time, this strategy could be favored in day-to-day clinical practice.

A preceding study of the APM research network was able to provide data on physical symptoms and psychosocial burden of 500 patients after the diagnosis of incurability by using an assessment strategy in a non-comparative longitudinal cohort study (12, 13). Patients in this preceding study showed quite variable symptom and distress levels, suggesting quite variable needs for supplementing specialized, multi-professional palliative care for some patients, and suggesting the usefulness of one kind of *screening/assessment* in order to detect patients in need (12, 13). The scope and perceived intensity of physical symptoms and psychosocial distress was comparable between the two studies.

Within the *screening* group, we found evidence that the quality of life according to IPOS can be maintained or even improved despite the course of the disease. The IPOS, which was periodically recorded in the *screening* arm, showed that the quality of life increased slightly. This may be explained, for instance, by the effect of palliative anti-cancer therapies (that began after enrollment by definition), by a response shift phenomenon related to increasing resilience towards the constraints of their illness (32), or by other positive factors such as successful palliative care interventions. A systematic review of quantitative studies suggests that resilience and hope, independence, social support, spirituality, fatigue, emotional distress, and coping skills are interrelated factors in patients with terminal illnesses. Prior experience with illness and life adversity, meaning-making, reconciling with life’s finiteness, acceptance of illness, control, and other factors for resilience were additionally found in qualitative studies (33). Another study points to the importance of medical communication, which can have a significant impact on the patient’s well-being and remaining life  (34). However, the questionnaires cannot provide clues on which reasons the patients themselves would attribute to an eventual improvement in their quality of life.

A significant improvement in QoL (psychological strains and physical symptoms, see **Table 3**) as per NCCN DT and IPOS was also observed within the *screening* group compared to baseline. In contrast, in the *assessment* group, significant improvement was only recorded in the IPOS subscore for psychological strains. This could indicate that low-threshold screening is better suited to record psychological stress and symptom stress and to react by the treating physicians. As there was insufficient data on palliative treatments or psycho-oncological care during the study, no precise statement to this end can be made.

On the other hand, the probability of hospitalization and the amount of days of hospitalization was higher in the *screening* group than in the *assessment* group. It is up for debate whether a regular screening encourages hospital stays or whether the patients' condition in the *screening* group was worse and therefore needed longer treatment.

## Strength and limitations

The study not only compares two distinct symptom recording strategies, but also provides more detailed insight into reported quality of life shortly after the diagnosis of incurable cancer. We demonstrated the variability in symptom spectrum and intensity, and provided data that the perceived quality of life may also improve even in advanced, eventually progressive disease. Due to the recruitment of a large number of study sites (24), some of them with specialization on few cancer entities, a large spectrum of different cancer entities could be included, and sub-group analyses are pending.

A major limitation of the study was the concurrent COVID-19 pandemic and its profound logistical implications, which severely impacted patient recruitment at many study sites. Study personnel were often not allowed to visit in-patients on a regular basis, and during the pandemic, staff resources were spent even more focused on patient care compared to study activities, and on-site study monitoring was not possible. Forwarding test results to clinical charts and utilization was likewise impaired by pandemic restrictions. Digital study monitoring would have eventually ensured data integrity by displaying prompts in case of missing input or errors even under pandemic study conditions. Especially smaller study sites were filled to personal capacity more quickly than larger facilities. Therefore, particularly data about the inclusion of palliative care and other emergency or supportive structures was insufficiently obtained, and no reasonable results on these proposed secondary objectives were gathered. Furthermore, the planned documentation of the reasons of patients not to participate was affected by the very special circumstances in the years 2020–22.

Another limitation of the study might be a potential learning bias by the recruiting physicians. Due to the nature of the study, there may have been a learning effect that could prompt recruiting physicians to ask patients more frequently about their well-being, regardless to which group the patients were assigned to. This cannot be prevented without allocating the various study sites to just one intervention arm. This idea, however, was discarded because the unequal structure of the institutions involved (university hospitals, medical practices, community clinics) would have made it difficult to compare the data.

The therapeutic consequences of the information gathered from the questionnaires for the further treatment of a patient (for instance, to refer to palliative or other supportive services) lay with the treating physicians. Since the study intervention focused on the recording of symptoms and needs only, no criteria were established as to how physicians should act in case of positive test results. This limitation may also have contributed to the fact that both arms exhibited a similar development in QoL.

## Conclusion

A comprehensive, multidimensional *assessment* did not significantly differ from low-threshold *screening* in preserving several dimensions of quality of life. Even if no significance level was reached in neither direction, it might be suggested that low-threshold, resource-saving, and easy-to-handle screening may be prioritized in day-to-day clinical practice. Survival rates did not differ significantly between the two groups. However, QoL scores had improved significantly by the end of the observation period in the *screening* arm. Further research is required to find out the reasons for this improvement in QoL and the associated reduction in distress and symptom burden. Like other studies, this study is constrained by the data available. Also, the combination of *screening/assessment* and resulting intervention (test-driven intervention) should be focused on in further trials.

Our findings may positively stimulate the implementation of structured screening programs for supportive and palliative care needs in certified cancer centers.

## Data availability statement

The raw data supporting the conclusions of this article will be made available by the authors, upon reasonable request.

## Ethics statement

The studies involving human participants were reviewed and approved by Ethics committee of the University Medical Center Göttingen. The patients/participants provided their written informed consent to participate in this study. The study protocol was approved by the ethics committees of all 24 study sites (PI study site no. 23/2/19) and followed the Declaration of Helsinki Ethical Principles for Medical Research. SCREBEL was registered in the German Registry for Clinical Studies (DRKS No. 00017774). Patients were included after written information, clarifications of the study and written consent.

## Author contributions

Manuscript writing: All authors. All authors contributed to the article and approved the submitted version.
